# Community health workers’ counseling is based on a deficit model of behavior change

**DOI:** 10.1371/journal.pgph.0004167

**Published:** 2025-07-23

**Authors:** Micah B. Goldwater, Faiz A. Hashmi, Sudipta Mondal, Cristine H. Legare

**Affiliations:** 1 School of Psychology, The University of Sydney, Sydney, New South Wales, Australia; 2 The Center for Applied Cognitive Science, The University of Texas at Austin, Austin, Texas, United States of America; 3 Project Concern International India, Delhi, India; University of Greenwich, UNITED KINGDOM OF GREAT BRITAIN AND NORTHERN IRELAND

## Abstract

In 2005, India launched the Accredited Social Health Activist (ASHA) program, which has augmented access to medical services and health education in marginalized rural communities. Despite notable progress in health delivery, uptake of medical services remains below target levels. The current research asked ASHAs and their clients why people reject medical advice and what the ASHAs could do to convince them otherwise. Our results identify a consistent mismatch between reasons to reject advice versus how to persuade clients to follow the advice. Two reasons were primarily cited for rejecting the uptake of medical services: insufficient or inaccurate understanding of the medical benefits of these services and the dynamics of the social situation, such as pressure from family members. In contrast, the predominant solutions addressed these knowledge gaps; ASHAs and their clients felt that highlighting the health advantages would be the most effective persuasion technique. ASHAs and their clients infrequently mentioned strategies addressing societal dynamics and norms. This mismatch between barriers to uptake and solutions suggests that the ASHA program inadvertently operates with a “deficit model” of decision-making and persuasion. The deficit model is the belief that the way to convince people to comply with health recommendations is to address their knowledge deficit by educating them on the medical benefits. The current research suggests that ASHAs should be trained in the science of belief revision and behavior change, which requires directly addressing the concerns and motivations of others, not just providing information.

## Author summary

In 2005, India started the ASHA program to help rural communities get better healthcare and health education access. While the program has improved health services, many do not follow medical recommendations. This study asked ASHAs and their clients why people refuse medical advice and how ASHAs could encourage them to follow it.

The study found two main reasons why people avoid using medical services. First, they do not fully understand the benefits of these services, or they believe incorrect information. Second, social factors, like family pressure, contribute to a lack of compliance. We found a disconnect between these reasons and the advice provided by ASHAs and clients who primarily suggested solutions that focused on educating people about the health benefits. They rarely mentioned strategies to deal with family or social pressures. There is a gap between why people refuse medical advice and how health educators should persuade them to follow it. This gap reveals a deficit assumption of reasoning that people need more information about health benefits to change their minds. Our research suggests that ASHAs need training in bridging the gap between client reticence to adopt recommended behaviors and the advice they provide. Instead of only giving information about health benefits, they need training to engage with underlying health beliefs and concerns.

## Introduction

Significant global advancements have been made in maternal and infant health, yet pressing gaps remain in the provision of health services, particularly in underserved areas. In India, disparities in health outcomes are pronounced, especially in states with large rural populations like Bihar. Over the last two decades, India has achieved a commendable 77% reduction in maternal and infant mortality rates (MMR and IMR). Yet, as of 2018–2020, Bihar’s MMR remains high at 97 per 100,000 live births, and the IMR stands at 35.2 per 1,000, indicating a pivotal need for enhanced healthcare interventions (Office of the Registrar General India, 2020; International Institute for Population Sciences- IIPS, 2021). Beyond survival, the health challenges persist through the pregnancy journey, with a staggering 63.5% of mothers in Bihar experiencing anemia and a mere 10.9% of children under two years of age receiving a diet that meets nutritional adequacy standards (IIPS, 2021).

One part of the government’s strategy to improve perinatal health outcomes was to create a community healthcare worker system. In this system, members of marginalized communities work to increase the uptake of biomedical services, acting as a bridge between their communities and government medical institutions. Specifically, the Indian government created the Accredited Social Health Activist (ASHA) community healthcare worker program. The ASHA program was launched in 2005 “as a key component of their National Rural Health Mission (NRHM, and now NHM) to strengthen rural government service delivery, as well as community engagement and ownership in health programs (see guidelines of roles and responsibilities (https://nhm.gov.in/images/pdf/communitisation/task-group-reports/guidelines-on-asha.pdf); training modules (https://nhm.gov.in/index1.php?lang=1&level=3&sublinkid=184&lid=257). The ASHA program involved the selection of one woman per village (approximately 1 per 1,000 population) who would receive an initial 23 days of training on basic health topics and link community members to health services, provide basic first aid and supplies, and mobilize the community around water, sanitation, nutrition, and health issues” [1]. The program’s core focus has been maternity and perinatal health [2].

ASHAs play a significant role in promoting the health of their communities, characterized by direct and family-focused interactions with their clients. These interactions are not limited to providing information but involve active engagements and sustained dialogues. The introduction of the ASHA program has ushered in improvements in multiple health behaviors, including antenatal checkups and institutional deliveries [2]. New research on ASHA efficacy underscores their pivotal role in influencing the health behaviors of their clients. For instance, a woman who frequently interacts with an ASHA and receives counseling is more likely to follow recommended perinatal health practices [3].

Despite notable successes in enhancing the accessibility and availability of critical health services and information, an unmet need remains. To align with the targets set under the Sustainable Development Goals (SDG) by the National Health Mission (NHM), we need to improve the levels of service uptake further [4–6]. There is a dearth of clear and actionable strategies required to continue improving the ASHA program’s impact beyond its current accomplishments [1].

Health messaging is one of the most common strategies employed in the ASHA program. Advocating for behavior based on risk reduction and health benefits is typically based on a “deficit model,” where health experts attribute rejection of medical advice to a lack of adequate scientific knowledge. This results in a noteworthy disconnect between the envisioned role of ASHA and the prevailing public health strategy for behavior change [7–9]. This model, built on unidirectional information transmission from experts to the public, is often at odds with the reality of health communication on the ground. Decades of research have consistently argued against the exclusive use of this model, demonstrating that the evaluation of information and trust establishment is strongly influenced by cultural values, social identity, and societal norms [10–15]. Yet, paradoxically, the deficit model remains deeply rooted in public health and science communication strategies [16–18], showing its inadequacy in inducing the desired changes in beliefs and behaviors [19]

Analytical methods, including surveys and brief educational interventions, often reveal that knowledge does not necessarily align linearly with attitudes or decisions [7,20–22]. Instead, information that resonates with recipients’ social identity and cultural values has a greater likelihood of impact [23,24]. This growing body of evidence indicates that the one-way transmission of knowledge, a key feature of the deficit model, may be fundamentally flawed [25]. In medicine, a shift towards shared decision-making has enhanced patients’ medical understanding and trust in their doctors and improved decision-making more significantly than mere information provision [13,26–30]. The efficacy of ASHAs’ counseling could be improved by moving beyond the deficit model and adopting an approach that engages with individuals’ values, beliefs, misgivings, fears, and concerns. Increasing the efficacy of behavioral change requires realigning public health strategies to integrate more effectively with ASHA’s envisioned role as a mediator of health knowledge and behavior change within communities.

## Current study

This study is a component of Project RISE, a mixed-methods research initiative to enhance maternal and newborn health in Bihar. It does so by creating tools that bolster the motivation and performance of ASHAs [3,28]. The project emerged partly due to a perceived mismatch between the original conception of ASHAs and their current functioning. Initially, ASHAs were envisaged as local intermediaries, building bridges between their communities and the public health system. They were also seen as catalysts for positive change in their communities, helping to spread and normalize scientifically validated health practices. Lastly, ASHAs were to extend health services to the local populace [31,32]. This vision meant ASHAs offered more than just medical services; they were to help foster a transformation in societal attitudes towards such practices and change the culture of medicine and health.

Understanding how they and their clients perceive and make health decisions is essential, considering influences like logic, biases, culture, and personal experiences to create impactful training for ASHAs. This insight can inform the design of tailored training content. The objective of the current study was to document what ASHAs and their clients identify as barriers to the uptake of recommended biomedical services and what ASHAs believe could persuade or support clients to increase the uptake of biomedically recommended behaviors. We did so by presenting vignettes of short fictional scenarios of clients either following or rejecting ASHA advice and eliciting possible explanations for why the characters did so by ASHAs and their clients. Because the current training structure primarily focuses on providing information about health benefits, we aimed to identify to what degree ASHAs focus on this strategy as a means for persuasion and to identify other insights their experience and knowledge offered.

Most research surrounding the ‘deficit model’ has primarily focused on interactions between experts and novices, groups often exhibiting significant disparities in knowledge, education, and potential cultural and social backgrounds [33,34]. In contrast, the present study delves into the subtleties of communication between ASHAs and their clients. This relationship is less distinctly stratified than that between an expert and a novice. ASHAs are members of their communities; they are all literate, and their training means they typically have more biomedical knowledge than their clients (many of whom are illiterate; see below). Nevertheless, the knowledge and cultural gaps in this context are narrower compared to the settings of prior research that investigated how medical and public health experts communicate with lay populations. Thus, ASHAs seem particularly likely to motivate their clients by using knowledge of their culture and social norms to engage in open dialogue rather than simply informing others of what is best for them. In contrast, because a deficit model of behavior change currently informs how ASHAs are trained to engage with their clients, they may rely on this training and act consistently with other health professionals seen across many healthcare contexts. Because ASHAs and CHWs more generally serve communities with poor population health outcomes, understanding how they attempt to deliver services to identify ways to improve outcomes is critical.

We examined how ASHAs and their clients (young mothers) reason about how women in the community make medical decisions and what ASHAs can do to influence these decisions. Our goals are to determine 1. whether ASHA operates with a (implicit) deficit model (primed by the health structure and training) where they think ASHAs can influence decisions by simply informing their clients of the health benefits of their recommendations and identify 2. what other means of persuasion or engagement they may be better able to leverage to increase service uptake. ASHA’s know their communities, the culture, and their clients well. However, our working hypothesis is that the prioritization of service extension over cultural facilitation and social change in ASHA training is based on a deficit model of behavior change. If all an ASHA is expected to do is link people with medical services, then it makes sense that they would try to do so by discussing the medical benefits of these services. Thus, we may see evidence that they do not use their rich cultural knowledge to persuade. Instead, they may persist in telling clients what is best for their health.

To investigate how ASHAs reason about the decision-making process, we developed a series of vignettes that described situations of young mothers in Bihar considering options for perinatal health practices. Health decisions are collective processes. Young mothers typically live with their husbands and families, and their mother-in-law is often a key influencer in perinatal and reproductive health practices. The entire household can be part of the decision-making, and cultural and religious beliefs, social norms, and prior experiences inform this process. These influences may not always align with the ASHA’s recommendations. The vignettes described the young women’s household situation and the ASHA’s recommendation, for example, to feed a young mother’s infant colostrum. There were two versions of each vignette, one where the mother did what the ASHA recommended and another where the mother did not. The vignettes were followed by a series of questions that elicited consideration of multiple aspects of the decision process from multiple perspectives, such as why the young mother made the decision, what she did, and what the ASHA could have done to persuade her differently.

Examining how they interpret and handle various situations is crucial to optimizing the influence of Accredited Social Health Activists (ASHAs) on their clients’ health decisions. One method involves presenting ASHAs with vignettes that depict either adherence or non-adherence to biomedical recommendations. For instance, the vignette might present a mother who either complies with (a ‘consistent’ condition, for example, gives birth at the hospital) or rejects (an ‘inconsistent’ condition, gives birth at home) ASHAs’ health recommendations. By manipulating these scenarios and encouraging ASHAs to consider multiple perspectives, we assessed the connection between their understanding of these situations and the knowledge they deem useful for persuasion. If ASHAs were operating under the deficit model, they would likely cite biological or health reasons more frequently when strategizing to persuade the mother in the vignette. This response pattern is compared to considering the mother’s reasons for her decision from other perspectives.

In addition to understanding what knowledge an ASHA may have that can be better applied, we examined to what extent her training and experience changed her perspective on these decisions compared to other community members. These other community members may offer further perspectives to leverage to increase medical service uptake. Thus, we also interviewed mothers who are the clients of ASHAs to make a comparison. ASHAs are also mothers, but the client-mothers are overall younger and without the ASHA’s training and experience.

The current study, which presents the data from vignettes about client-mother’s decisions, had a 2 (Client decisions: Consistent vs. Inconsistent with ASHA advice) X 2 (Respondent: ASHAs vs. Mothers) design. Our primary research questions were:

1. Is there a mismatch between respondents’ reasons why a client would reject an ASHA’s advice and what ASHAs should do to convince them to follow it?
2. Do different health decisions (such as giving birth in a clinic or vaccinating an infant) elicit different kinds of reasons and means of persuasion?

We predicted that the reasons for the client’s decisions would reflect many factors. These include the perceived health benefits or negative side effects of biomedical practices, the social dynamics of the clients’ households (both in support or against ASHA advice), and other forms of incentives and barriers (such as payment related to giving birth in a clinic, or trouble with transportation to a clinic, respectively). Notably, we predicted that strategies suggested for how AHAs could persuade their clients would have a smaller range of factors. If acting with an information deficit model, the suggestions for persuasion would focus on health benefits. We did not have strong directional hypotheses for the analyses comparing different specific health behaviors in terms of these broader categories, such as focusing on health benefits versus the social dynamics of the household. However, exploring these patterns across different decisions is crucial to understanding how to improve ASHA training moving forward.

## Methods

### Ethics statement

This project’s methodologies, surveys, and consent procedures were reviewed and approved by the Institutional Review Board of the University of Texas at Austin (Study Number: 2018-01-0027; Approval Date: Feb/23/2018) and by Sigma Institutional Review Board in India (Study Number: 10056/IRB/D/18–19, Approval Date: Dec/22/2018). Participation in this study was strictly voluntary, and informed consent was obtained from each respondent before they provided their responses about the vignettes. The consent included a brief description of the vignettes, a description of the respondent’s role in the study, including the expected duration of the respondent’s participation, a clear indication that participation is voluntary and that the information provided would be confidential*.* Each interviewer obtained consent verbally and recorded it as a response on the form. Interviews with research participants were conducted between September 25th and October 5th, 2019.

Here, we report just the aspects of the methods relevant to the current research questions. We have information about these participants that is not currently relevant, and they were asked several other questions, including the vignettes discussed here and other topics beyond the scope of this paper.

### Participants

146 ASHAs and 291 young mothers in Bihar, India, responded to interview questions concerning the decision vignettes as part of Project RISE. Of the 146 ASHAs, we have demographic information for 131. They had a mean age of 38 years (range 23–65). They were all literate as per the job requirements. In terms of the caste distribution, 92 ASHAs were from Other Backward Class (OBC), 21 from the General Caste (GC),16 from Scheduled Caste (SC), and 2 ASHAs were from Scheduled Tribe (ST) categories. Of the 291 young mothers, we have demographic information for 211. They had a mean age of 23 years (range 16–41) and a mean age-married of 17 years (range 12 – 25). 102 of 211 were literate. Regarding the caste distribution, 133 mothers were from the OBC category, 53 were from GC, 19 were from SC, and 6 were from the ST category (Because of the substantial number of participants with missing demographics, they are not part of the regression models below; including demographics reduces our statistical power. However, patterns of results were qualitatively the same in analyses that included demographics; not reported here.).

### Sampling frame and method

The vignette sampling was informed by a quantitative survey conducted earlier across four districts in Bihar: East Champaran, Samastipur, Gaya, and Purnia [3]. During the quantitative survey, a list-based framework was established by compiling a list of mothers with children aged 0–6 months from the ASHAs in each Anganwadi Center (AWC). From this list, three mothers were randomly selected per AWC using a random number-generating application on the data supervisors’ phones. 400 ASHAs and 1,200 mothers were surveyed in the quantitative phase.

For the current study, two districts—East Champaran and Purnia—were selected for their distinct sociocultural characteristics. 150 AWCs were randomly chosen from these districts from the initial sampling frame. From each AWC, one ASHA and two mothers (with children aged 0–2 months) were selected, yielding a sample of 150 ASHAs and 300 mothers. This approach ensured a combination of list-based frameworks (through the initial mother listings) and geographical frameworks (covering a diverse set of districts and AWCs).

### Materials

Eight vignettes shared the same structure. Each vignette presented a “young woman in Bihar” who needed to decide about a perinatal health practice recommended by her ASHA. The vignette topics were feeding her newborn colostrum, exclusive breastfeeding for her infant, taking iron/folic acid (IFA) tablets during pregnancy, getting vaccinated during pregnancy, getting her infant vaccinated, family planning (i.e., taking birth control) before having her first child, family planning after her second child, and giving birth at a clinic rather than in the home.

The vignettes started with a brief setup. For example.

*Rekha is a woman in Bihar. She is three months pregnant with her second child. She lives with her husband, her in-laws, and her child. Her ASHA encouraged her to take IFA tablets during pregnancy.*

There were two versions of each vignette: the Consistent version, where the young mother made a decision consistent with the ASHA’s recommendation, and the Inconsistent version, where the young mother made a decision inconsistent with the recommendation. For example:

Consistent: *Rekha decided to take IFA tablets*

Inconsistent: *Rekha decided not to take IFA tablets*.

The vignettes were followed by a series of questions in the same order. We focus on two questions here, Questions 1 and 2 (see S1 Table for the complete list and sequence). Question 1 asked why the young mother made the decision that she did. For example-

Question 1 Consistent: *Why do you think Rekha chose to take IFA tablets?*.

Question 1 Inconsistent: *Why do you think Rekha chose not to take IFA tablets?*

Question 2 first noted the disagreement between the ASHA and the other member of the household and then asked, in the Consistent condition, what did the ASHA say to convince the young mother to follow her recommendation, or in the Inconsistent condition, what could have the ASHA done differently. For example-

Question 2 Consistent: *Rekha followed her ASHA’s recommendation and took IFA tablets. Rekha’s husband disagreed with Rekha’s ASHA and told Rekha not to take IFA tablets. What do you think the ASHA said about convincing Rekha to take the IFA tablets?*

Question 2 Inconsistent: *Rekha did not follow her ASHA’s recommendation to take IFA tablets. Rekha’s husband disagreed with Rekha’s ASHA and told Rekha not to take IFA tablets. What do you think the ASHA should have said to convince Rekha to take the IFA tablets?*

### Procedure

Young mothers and ASHAs were interviewed at their homes by 14 female field investigators and 3 data collection supervisors. The data collection took 13 days, and the field investigators were trained for two additional days in-house. Interviews were audio-recorded and supplemented with detailed field notes.

Every participant responded to four of the eight vignettes (two in the Consistent condition and two in the Inconsistent condition). This was counterbalanced across participants, so each vignette was discussed roughly the same number of times in each condition as the others. The investigators completed transcriptions. Translations were outsourced to an external agency.

### Design summary & predictions

There was a 2, Decision Condition (Consistent vs. Inconsistent) X 2, Respondent (ASHA vs. Mother) X 2 Vignette Question (1. Decision reasons vs. 2. Influence methods) design. The key dependent variables were whether the respondents gave reasons based on health and biology or focused on other aspects, such as the social dynamics of the household. These codes are elaborated below.

Vignette Question 1 asked why clients either follow or reject ASHA advice. Vignette Question 2 asked what convinces clients to follow or reject ASHA advice. Differences in the patterns of responding between Vignette Questions 1 and 2 provide answers to Primary Research Question 1. If Question 2 elicits more health-centered responses and fewer responses based on the social situation than Question 1, that suggests the respondents are operating with a deficit model of medical decision-making.

### Coding

Interviews were recorded in Hindi and translated into English for coding and analysis. These interviews were also recorded on digital tape and used during the translation. The vignette coding had two steps. Project RISE is interested in characterizing the specifics of these eight medical decisions described in the vignettes and capturing more general principles that apply across scenarios. The latter is relevant to the current paper. A coding scheme was first created for each specific scenario (not discussed here). Four broad categories of responses were developed to generalize across the diverse set of vignettes that subsumed these more specific codes. The first is *Health-Biology*, which indicates that the character in the vignette decided based on something related to their health or cited something about biology. This could include information about specific biological or health benefits or a more general discussion of health, strength, or illness.

Specific health reasons:

*Rekha decided to eat the IFA pill because it creates blood in the body, keeping the mother and baby healthy. Eating the IFA pill will not cause swelling in the hands and legs, the baby will be healthy in the womb, and tiredness, weakness, or dizziness will not occur.*

More general:

*Rekha decided to take the IFA pill so that both mother and child are healthy.*

Our primary analyses consider specific and general reasons, but we also analyze the specific responses as a proxy for richer medical knowledge.

The second category was *Social Dynamics*. This covered specific social interactions, social priorities for the mother in the story, or broader social norms.

Specific interactions:

*Anita won’t listen to her mother and mother-in-law and will feed colostrum to her child*.

Social priorities:

*“Rinjani would not want to have a child for two to three years right now, she would want to live free now…”*

Broader social norms:

*Earlier, no one used to get the vaccine, but now everyone gets it.*

A third category was *Other Benefits-Costs*, which, for example, covered financial incentives, logistical issues, and administrative benefits.

*The mother gets money for delivery. Also, the child can get their birth certificate. But at home, women and children do not get any kind of facility like this. All Below Poverty Line (BPL) families get insurance for delivery in the hospital.*

The fourth category was *knowledge ignorance*, which focused on the mother’s knowledge, experience, or beliefs in the story.

*Neetu decided not to start using the means of family planning as she was not aware of the fact that she was afraid.*

These codes are not mutually exclusive (more examples are provided in S2 Table). The questions were open-ended so that respondents could give many reasons. For example, the complete response of an excerpt above shows that it follows both a Health/Biology and a Social Dynamics response.

*Suman must have realized that if she gets the TT vaccination, she will be prevented from tetanus, and she and her newborn will stay healthy. Earlier, no one used to get the vaccine, but now everyone does.*

Because any response could have any number of coding categories, typical ways of calculating interrater reliability would not apply as they assume mutually exclusive code categories. Some metrics count that not using the same code is a sign of agreement, but some codes were rare (e.g., Knowledge/Ignorance for Question 2 were under 1% of responses), so simply not using those rare codes would inflate agreement levels. Instead, we did the following.

Two raters coded the same 157 responses. Agreement between the two raters was defined by points earned per response. Perfect agreement earned 1 point. No agreement earned 0 points. The rater who checked more code categories was defined as the standard for each response under consideration. Then, the points that the response earned were the proportion or the codes the second rater coded the same as the standard. If both raters only had 1 code category checked, and it was the same code, they earned 1 point; if it were different, 0 points. If one rater checked two categories, and the other did both the same, that is 1 point. If one was the same and different, that earned.5 points. If the second rater only checked one code, and it agreed with one of the two the original rater checked, that was also.5 points. If the standard rater checked three codes, that response could earn 0,.33,.67, or 1 point. Out of 157 maximum points for the 157 responses, the two raters earned 147.5, or 94%.

## Results

### The pattern of responses across eight health behavior vignettes

We first examined the pattern for each vignette. Table 1 presents the four categories of responses for the eight vignettes for Question 1 (reasons to decide). Health-biology is the primary reason category given for every health behavior (at least 69% of responses included a health reason) except for Family Planning after the mother has multiple children (which elicited responses that mentioned health benefits at a rate of only 27%). This means that after a woman has had multiple children, the respondents are likely to focus on lifestyle and economic factors as reasons to consider having more children. This item had the largest proportion of “Other benefits” responses (86%). Only two other behaviors elicited over 2% of responses that included “Other benefits,” such as family planning when the mother had yet to have any children (35%) and institutional delivery (63%). However, unlike Family Planning when the mother had multiple children, these also elicited a high proportion of responses that mentioned health benefits (69% and 73%, respectively). To foreshadow some analyses below, we will group the three health behaviors that elicited over 30% Other Benefits/Costs responses into a category and grouped the other five into another to test whether the Independent Variables (Condition, Question, & Respondent) have the same effects for both categories of behaviors.

**Table 1:** Results of vignette question 1 (collapsing across Condition and Respondent) for each vignette.

Vignette	Health-Biology	Social Dynamics	Other Benefits- Costs	Knowledge – Ignorance
Colostrum	0.89	0.17	0.00	0.18
IFA	0.92	0.12	0.00	0.15
Exclusive Breastfeeding	0.87	0.11	0.02	0.14
Vaccines – Pregnancy	0.75	0.12	0.00	0.23
Vaccines – Infancy	0.94	0.05	0.02	0.09
Family Planning 1 – no children	0.69	0.24	0.35	0.08
Family Planning 2 – multiple children	0.27	0.14	0.86	0.06
Institutional Delivery	0.73	0.12	0.63	0.28

Next, we will present the proportions of responses per each health behavior relevant to addressing our primary research question: are there differences between the kinds of reasons respondents offer for rejecting ASHAs’ advice and what an ASHA can do to convince a client to follow their advice? In S3 Table we present all four categories of responses for the eight health behaviors broken down by respondents and conditions for both Vignette Questions (concerning 1. decision reasons and 2. influence methods). Here, we discuss the most critical comparisons. Table 2 presents the Heath-Biology and Social Dynamics responses for the Inconsistent condition (when the client rejects the ASHA’s advice), broken down by Respondent (ASHA vs. Mothers) for all eight health behaviors.

**Table 2:** Comparing Q1 and Q2 of the Inconsistent Condition for Health-Biology and Social Dynamic Responses for ASHAs and Mothers for all eight health behavior vignettes.

Vignette	Respondent	Q1: Health	Q2: Health	Q1: Social	Q2: Social
Colostrum	ASHA	0.70	0.96	0.30	0.08
IFA	ASHA	0.76	0.98	0.21	0.1
Exclusive Breastfeeding	ASHA	0.80	0.67	0.15	0.1
Vaccines - Pregnancy	ASHA	0.33	0.98	0.31	0.12
Vaccines - Infancy	ASHA	0.85	0.96	0.15	0.2
Family Planning 1 - no children	ASHA	0.52	0.98	0.64	0
Family Planning 2 - multiple children	ASHA	0.25	0.33	0.71	0.19
Institutional Delivery	ASHA	0.46	0.88	0.42	0
Colostrum	Mother	0.81	0.9	0.13	0.01
IFA	Mother	0.90	0.94	0.14	0.03
Exclusive Breastfeeding	Mother	0.67	0.93	0.13	0.01
Vaccines - Pregnancy	Mother	0.52	0.9	0.30	0.05
Vaccines - Infancy	Mother	0.85	1	0.04	0.03
Family Planning 1 - no children	Mother	0.41	0.95	0.63	0.02
Family Planning 2 - multiple children	Mother	0.57	0.32	0.14	0.24
Institutional Delivery	Mother	0.42	0.91	0.18	0

To foreshadow the results of the inferential statistics, across the eight vignettes, the results are consistent with both ASHAs and mothers operating with an information deficit model. There were more responses concerning health benefits when answering how an ASHA could convince her client (Q2) to follow her advice, compared to why her client rejected her advice (Q1) for 7 out of 8 health behaviors (for both ASHAs and mothers, respectively). In contrast, for 7 out of 8 health behaviors, fewer responses discussed social dynamics when giving persuasion advice (Q2) compared to reasons for rejection (Q1; again, for both ASHAs and Mothers, respectively).

*Analyses to Answer Research Question 1: Is there a mismatch between the reasons respondents offer for why a client would reject an ASHA’s advice and what ASHAs should do to convince them to follow it?*

To answer this question, analyses are conducted in three steps.

Step 1. Analyze the reasons offered to explain client decisions in the responses to Vignette Question 1.

Step 2. Analyze the methods of persuasion recommended to ASHAs in the responses to Vignette Question 2.

Step 3- Identify mismatches between the reasons for decisions and the recommended methods of persuasion by contrasting responses to Vignette Question 1 with Vignette Question 2.

For our statistical analyses, to understand the effects of our independent variables (Client Decisions: Consistent with ASHA advice v. Inconsistent; Respondent: ASHA v. Mother), we generalized across the specific health behaviors (power would be reduced if individual vignettes were analyzed).

We conducted a series of mixed-effects logistic regression analyses. Each model focuses on a different response category, with a binary DV because the respondent either provided that response or did not. For example, we conducted separate analyses for Health-Biology responses than Social Dynamics responses. All models included random intercepts for Vignette (the specific health behavior) and Participant. For analyzing Vignette Questions 1 and 2 independently of each other in Step 1 and Step 2, respectively, their models contained fixed factors of Respondent, Condition, and their interactions. The models comparing the two vignette questions further included two-way interactions between Respondent, Condition, and Question (respectively). For the complete data set used in these analyses, see https://osf.io/hq9nu/?view_only=53543783f49c4fbe830283665593bd81.

***Analysis Step 1: The reasons why clients behaved consistently (followed) or inconsistently with (rejected) the ASHA advice, according to respondents (mothers and ASHAs).***

We analyzed responses to Vignette Question 1. We examined how the Consistent and Inconsistent conditions elicited different explanations from Mothers and ASHAs. Table 3 shows the proportions of each category of response. Table 4 summarizes the key findings by showing the statistically significant odds ratios from the mixed models (see S4 Table for fixed-effect parameter estimates).

**Table 3:** Proportion of responses of each category for Vignette Question 1.

Respondent	Condition	Health- Biology	Specific Health	Social Dynamics	Other Benefits-Costs	Knowledge - Ignorance
ASHA	Consistent	0.84	0.46	0.12	0.21	0.05
Mother	Consistent	0.86	0.31	0.05	0.19	0.00
ASHA	Inconsistent	0.59	0.25	0.36	0.18	0.37
Mother	Inconsistent	0.64	0.25	0.21	0.20	0.26

**Table 4:** Summary of effects for Vignette Question 1. Odds ratios- exp(B)- for fixed effect estimates with p < .05 are included.

**Effect**	**Health- Biology**	**Specific Health**	**Social Dynamics**	**Other Benefits- Costs**	**Knowledge- Ignorance**
Consistent - Inconsistent	9.671***	2.207***	0.312***		0.032***
ASHA - Mother		1.796***	2.244***		3.474***
Condition X Respondent		2.013**	1.986*	2.629*	6.706**

* p < .05; ** p < .01; ***p < .001

A Health-Biology reason had 9.67 times greater odds of being offered when the vignette character decided to be consistent with the ASHA’s recommendation than when she went against the recommendation. In contrast, when explaining why the character went against the ASHA, a Social Dynamics response had 3.21 times greater odds, and a Knowledge-Ignorance response had 31.25 times greater odds of being offered compared to the Consistent condition (the reciprocals of.312 and.032 as shown in Table 4). ASHAs had 2.24 times and 3.47 times greater odds of offering a Social Dynamics and Knowledge-Ignorance response (respectively) than the mothers. These differences interacted with the condition, with the ASHA’s greater odds than Mothers to offer a Social Dynamics and Knowledge-Ignorance response was greater for the Consistent condition than the Inconsistent (*exp(B)* = 1.99; *exp(B)* = 6.71, respectively). Highlighting how ASHAs have richer medical knowledge than their clients (they are both older and have higher literacy rates, in addition to the ASHA training; see above), they were more likely to offer a specific health reason (*exp(B*) = 1.80), even though they were not significantly more likely to offer a health reason overall. This ASHA advantage effect interacted with condition (*exp(B*) = 2.01) because ASHAs only offered more specific reasons in the Consistent condition.

In sum, the respondents indicated that mothers followed what the ASHA says because of the mother and child’s health. However, a mother may often go against the recommendation because of social reasons or ignorance. Further, there are health-related reasons to go against the advice, but often these are based on misconceptions. One example:

*Suman’s mother-in-law says that earlier, people would not take the injection, so they would not have had a child. The Mother-in-law says that the injection will damage the child.*

Consistent with the deficit model, false belief and ignorance were offered as reasons to oppose the ASHA. However, the Social Dynamics responses show a greater understanding of all the factors that can affect medical decision-making. Vignette Question 2 examines whether the respondents believe these social factors can be used to persuade someone who may otherwise be resistant to following the ASHA’s advice.

***Analysis Step 2: The methods of persuasion that the ASHA in the vignette used successfully (in the Consistent condition) or could have used (in the Inconsistent condition) to convince their clients to follow their advice, according to respondents (mothers and ASHAs).***

Table 5 shows the proportions of each response to Vignette Question 2, and Table 6 shows the statistically significant odds ratios (see S4 Table for more model information). Because Knowledge-Ignorance responses were so rare, they were not analyzed with a mixed-effect model. These tables show that, again, the Consistent condition elicited more Health-Biology responses (*exp(B)* = 2.40), but it also elicited more Social Dynamics Responses (*exp(B)* = 2.60).

**Table 5:** Proportion of responses of each category for Vignette Question 2.

Respondent	Condition	Health Biology	Social Dynamics	Other Benefits Costs	Knowledge Ignorance
ASHA	Consistent	0.93	0.18	0.17	0.01
Mother	Consistent	0.90	0.12	0.21	0.00
ASHA	Inconsistent	0.84	0.10	0.24	0.01
Mother	Inconsistent	0.86	0.05	0.21	0.01

**Table 6:** Summary of effects for Vignette Question 2. Odds ratios- exp(B) for fixed effect estimates with p < .05 are included.

Effect	Health Biology	Social Dynamics	Other Benefits Costs
Consistent - Inconsistent	2.400***	2.598***	
ASHA – Mother		2.030***	2.043*
Condition X Respondent			

* p < .05; ** p < .01; ***p < .001.

***Analysis Step 3: Identify mismatches between what respondents say an ASHA can do to convince a client and why respondents offer to explain clients’ decisions to follow or reject an ASHA’s advice.***

**Fig 1:**
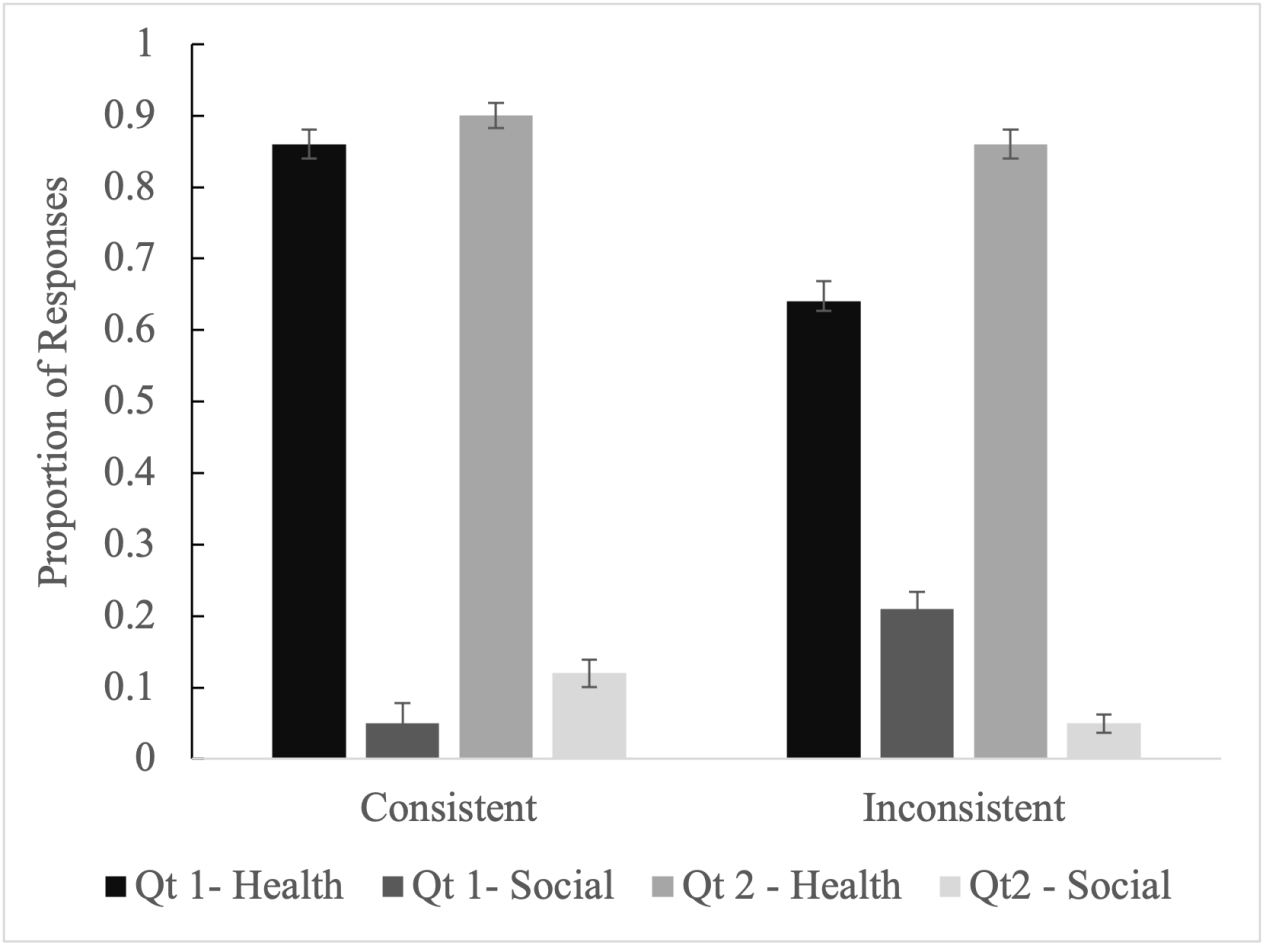
Proportion of responses, and standard error of proportions for ASHAs.

**Fig 2:**
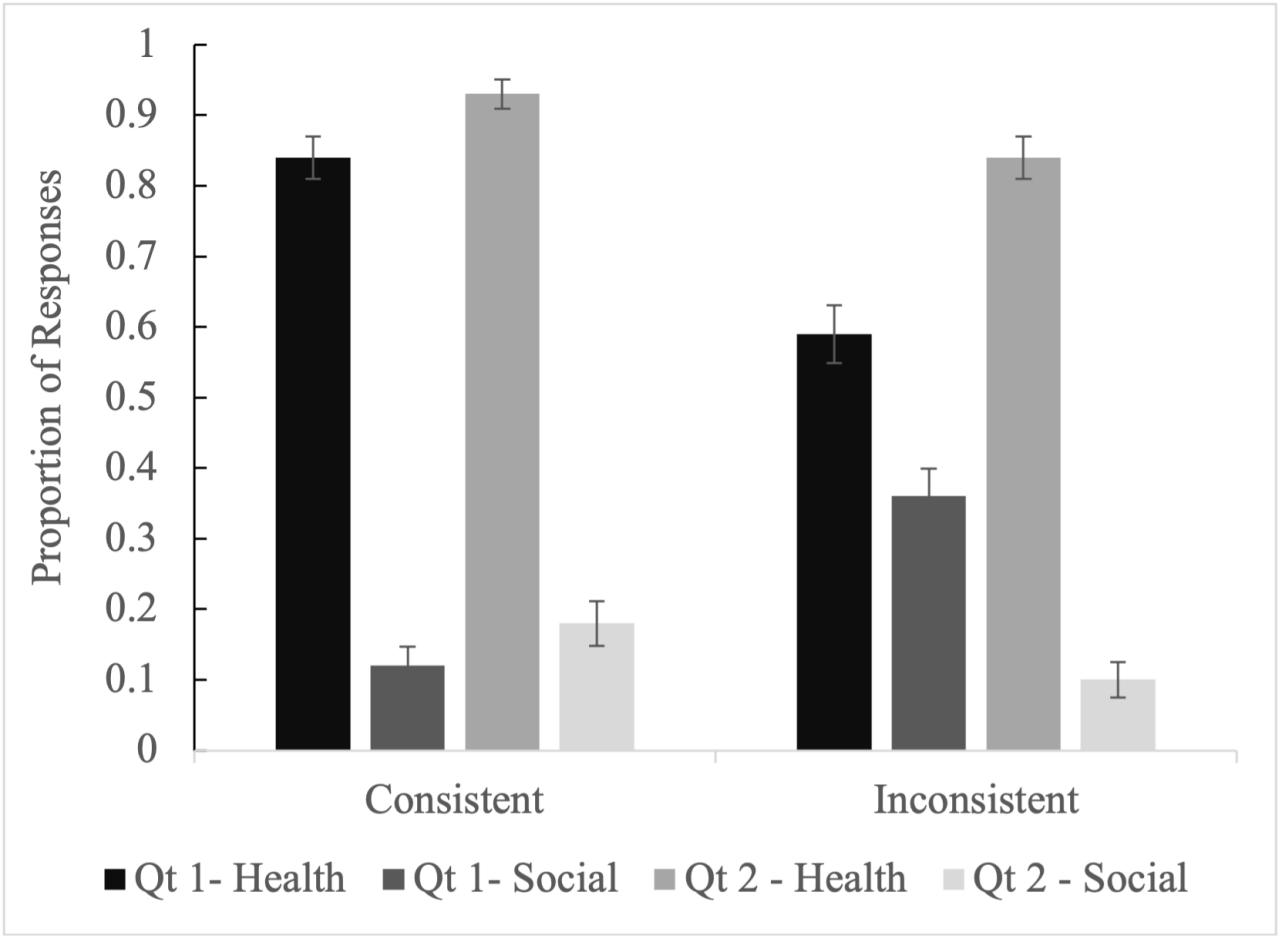
Proportion of responses, and standard error of proportions for Mothers.

Step 3 directly evaluates the use of a deficit model by contrasting responses to Vignette Questions 1 and 2; see Tables 7 and 8 and Fig 1 and Fig 2 (and S4 Table for more model information). These have fixed factors of Condition, Respondent, and Question and two-way interactions between Condition and Question and Respondent and Question. Given the above models, interactions between Condition and Respondent did not add important new information. As mentioned above, these models had random intercepts for Vignette and Participant. Here, we focus on Health-Biology and Social Dynamics as they are central to our research aims.

**Table 7:** Summary of effects of contrasting Vignette Question 2 and Question 1. Odds ratios- exp(B)- for fixed effect estimates with p < .05 are included.

Effect	Health-Biology	Social Dynamics
Consistent - Inconsistent	4.718***	
ASHA - Mother		1.867***
Question: 2–1	3.375***	0.682**
Condition X Question	0.242***	7.935***
Respondent X Question	1.764*	

* p < .05; ** p < .01; ***p < .001.

**Table 8:** Summary of simple effects for contrasting Vignette Question 2 and Question 1. Odds ratios- exp(B) for p < .05 are included.

Condition/Respondent	Contrast	Health-Biology	Social Dynamics
Consistent	2 - 1	1.66*	1.920***
Inconsistent	2 - 1	6.86***	.242***
ASHA	2 - 1	4.48***	0.6**
Mother	2 - 1	2.54***	

* p < .05; ** p < .01; ***p < .001.

The contrast between Questions 2 (which asks how ASHAs persuade their beneficiaries) and 1 (which asks why their beneficiaries made the decision they did) is consistent with ASHAs operating with a deficit model of medical decision-making. Most importantly, there are interactions between Condition and Question for both Health-Biology and Social Dynamics responses, and the Simple Effects (Table 8) show the pattern explaining the interactions. Respondents had 6.86 times greater odds of giving a Health-Biology explanation when indicating what could convince a vignette character to follow ASHA’s advice in Question 2 compared to why they rejected it in Question 1. In contrast, they had 4.13 times greater odds of giving a Social Dynamic response when explaining why they rejected the ASHA’s advice in Question 1 compared to what could convince them in Question 2(the reciprocal of.242 in Table 8). The greater reliance on Health-Biology reasons for Question 2 rather than Question 1 was true for both populations of respondents, but it was even more pronounced in ASHAs (*exp(B)* = 4.48) than in Mothers (*exp(B)* = 2.54). Likewise, ASHA specifically showed a significant reduction in Social Dynamics responses for Question 2 compared to 1 (*exp(B)* = 0.60). Still, mothers did not show this statistically significant reduction. Notably, there was no significant interaction between the Question and Respondent for Social Dynamics responses, as there was for Health responses. Thus, overall, the pattern is consistent with both mothers and ASHAs operating with a deficit model of behavior change, but ASHAs have a more pronounced model. This suggests that ASHA training does not induce a deficit model but that it potentially reinforces the default way people reason about medical decision-making.

Figs 1 and 2 visualize the interactions between Consistency, Question (Qt) and Response category. When explaining why medical advice is rejected (Inconsistent Qt1), Health responses are lower and Social responses are higher than when explaining how to overcome this rejection (Inconsistent Qt2), or explaining why beneficiaries accept medical advice (Consistent Qt1).

*Research question 2: Do different health decisions (such as giving birth in a clinic or vaccinating an infant) elicit different categories of reasons?*

To answer this question, we refer to the discussion above and Tables 1 and 2, and for deeper insights into the specific decision-making contexts, we analyze differences between the vignettes. Table 1 presents results from Q1, which shows a clear difference between vignettes the Other Benefits-Costs response is almost entirely due to 3 of the 8 vignettes-: 2 for family planning and 1 for institutional delivery. In turn, these three show fewer Health-Biology responses. Here, we present analyses akin to the analyses above but with an additional categorical variable-: Health vignettes (colostrum, breastfeeding, IFA tablets, and both vaccines) vs. Other vignettes (both family planning and institutional delivery).

There are many ways to examine differences between these two categories of vignettes, potentially, so here we stick to the critical analyses needed to answer our primary research question relating to the mismatch of responses given between vignette questions 1 and 2 (which is the key pattern concerning deficit-models of persuasion). The primary question is whether the increase in Health-Biology responses and decrease in Social-Dynamics responses is specific to one vignette category (collapsing across respondents). Table 9 shows the frequencies of the different responses, and Table 10 shows the relevant results from mixed-effects logistic regressions. These analyses contained fixed Vignette Category, Condition, and Question factors, with all two-way interactions and random intercepts of Vignette and Participant. Three-way interactions are not included because they did not improve the DV model fit. Table 10 focuses on the simple effects of contrasting questions 1 and 2, which best illustrate the potential consistency across categories, examining the critical question differences. S4 Table presents the full fixed-effect parameter estimate information.

**Table 9:** Proportions of responses further broken down by vignette category.

Vignette Category	Vignette Question	Condition	Health-Biology	Social Dynamics	Other Benefits- Costs
Health	1:	Consistent	0.97	0.07	0.01
		Inconsistent	0.76	0.16	0.01
	2	Consistent	0.96	0.16	0.03
		Inconsistent	0.93	0.06	0.01
Other Benefits	1	Consistent	0.75	0.08	0.54
		Inconsistent	0.40	0.27	0.65
	2	Consistent	0.84	0.08	0.49
		Inconsistent	0.72	0.08	0.55

**Table 10:** Summary of simple effects for contrasting Question 2 and Question 1 for both vignette categories. Odds ratios- exp(B) for p < .05 are included.

Vignette Category	Condition	Contrast	Health-Biology	Social Dynamics	Other Benefits- Costs
Health	Consistent	2 - 1		2.416***	3.44**
	Inconsistent	2 - 1	4.231***	0.330***	
Other Benefits	Consistent	2 - 1	2.067**		
	Inconsistent	2 - 1	9.902***	0.165***	.442***

* p < .05; ** p < .01; ***p < .001.

The Inconsistent condition is most critical for evaluating the use of deficit models. Here, both Health vignettes (*exp(B)* = 4.84) and Other Benefits vignettes (*exp(B)* = 8.50) elicit more Health-Biology responses and fewer Social Dynamics responses (Health: *exp(B)* =.29; Other Benefits: *exp(B)* =.20) for Question 2 than Question 1. In addition, for the Other Benefits vignettes, even the mention of these Other Benefits is reduced in Question 2 to persuade a client who is going against an ASHA’s recommendation, compared to Question 1 (*exp(B)* =.45).

## General discussion

Our objective was to examine the extent to which health-related decision-making among healthcare workers (ASHAs) and their clients (mothers) is based on a deficit model of behavior change. Using a vignette design, we asked ASHAs and clients to explain why people adopted or rejected biomedical recommendations and what ASHAs either did or could do better to convince them to adopt them.

First, we examined whether there is a mismatch between the reasons ASHAs and mothers offered for why a client would reject an ASHA’s advice and what ASHAs should do to convince them to follow their advice. Next, we examined whether different health decisions (such as giving birth in a clinic or vaccinating an infant) elicited different reasons and recommended means of persuasion. To interpret the answers to these questions, we compared their responses to the predictions of a deficit model of persuasion. If respondents relied on a deficit model, they would emphasize the importance of communicating the health benefits of the recommended medical practice to persuade, regardless of the potential reasons why the recommended practice was rejected.

To answer research question 1, we compared reasons for rejection with recommended methods of persuasion. We found that the most cited reason for rejecting biomedically recommended behaviors concerned the health of the mother and child (even if these were misconceived or inaccurate reasons). Likewise, the most recommended method of persuasion was to convince the client that the ASHAs medical advice was truly best for the client and their child’s health, for example, that vaccinating infants prevents them from getting sick. This suggests some alignment or “match” between why clients reject advice and what ASHAs should say to persuade them. This finding is consistent with a deficit model because of the centrality of health beliefs in determining decisions and the need to communicate the true benefits of recommended medical practices.

There was also evidence of a mismatch between why clients reject advice and what ASHAs should say to persuade them, which is consistent with a deficit model. When explaining why clients might reject an ASHA’s advice, respondents frequently cited social dynamics, including specific social pressures to take kinds of advice or broader social norms in the community. However, when asked how to persuade a reluctant client, responses referring to these social dynamic responses were significantly rarer. Thus, even though health beliefs were the most common reason for medical decisions, social dynamics also affected these decisions. Notably, the means of persuasion only reflected health concerns. This mismatch between reasons for rejecting medical advice and means of persuasion for both populations of participants, the ASHAs and the mother-clients, suggests both populations demonstrated reliance on a deficit model. This effect was more pronounced among ASHAs, suggesting that current ASHA training might inadvertently reinforce rather than challenge a deficit-oriented approach to behavior change. Training that focuses heavily on the benefits of medical recommendations, without equal attention to the psychosocial complexities that shape health behaviors, could lead ASHAs to prioritize knowledge-based persuasion techniques over strategies that address other real-world barriers and social motivations (Responses may have been biased towards a deficit model because this is how respondents think ASHAs should advise, even if the respondents think there are other ways to persuade. Even if true, the recommendation to move ASHA training away from this model would be the same.).

The focus on health benefits in these experiments may have been greater than what they would be in actual interactions between ASHAs and their beneficiaries. Here, the respondents’ focus may have reflected the information most available to them, aligning with the concept of the availability heuristic [35]. This is consistent with the result that health responses were the most frequent for both questions, respondent groups, and in both conditions. However, this does not explain the shifts in the frequency of health and social responses across questions, respondents, and conditions, given the experimental context and health focus remained constant. These shifts suggest that the deficit model—highlighting how gaps in knowledge shape responses—played a significant role in driving participants’ answers. Further studies could address this limitation by varying the information available in vignettes and comparing results to observational studies of these decision contexts in the real world.

We also examined whether different health behaviors described in the vignettes elicited different reasons for rejection and recommended means of persuasion. Three of the eight vignettes (giving birth at a medical institution and the two that concerned family planning) found a significant proportion of *Other Benefits/Costs* responses. These responses highlighted non-medical considerations like economic constraints, family preferences, and facility access. This finding highlights that factors beyond immediate health benefits and potential risks might more heavily influence certain health behaviors. However, these three vignettes all revealed evidence of the same kind of mismatches discussed above between reasons for rejection and recommended methods of persuasion. Regardless of how frequently vignettes elicited these *Other Benefits/Costs* reasons, this did not affect the fact that both ASHAs and mothers were less likely to cite *Social Dynamics* in their recommended persuasion methods than when discussing reasons for rejecting ASHA advice. Thus, there was evidence for a deficit decision-making model across different vignettes.

ASHA training focuses on medical knowledge, general health benefits, and navigating public medical institutions. Although ASHAs have facilitated the uptake of medical services, the training does not *sufficientl*y equip ASHAs with effective persuasion strategies, tools for initiating behavior change, or insights into emotional or motivational processes [1–6]. ASHAs should be trained to tailor their communication approach to specific health behaviors. For behaviors where social and logistical factors play a more prominent role, ASHAs must emphasize these aspects and develop strategies for overcoming potential obstacles. For example, training does not teach how to navigate the social dynamics of the family nor how to improve the quality of the dialogue (interpersonal communication) between the ASHA and her clients so that the clients may become more open to advice. These appeals align well with recent research on promoting science-backed decisions [36].

ASHAs have inherent strengths as women, mothers, and members of the communities they serve who possess shared sociocultural and individual experiences [37]. However, the training leaves ASHAs unprepared to incorporate these essential sociocultural aspects into their work. This approach has caused a shift in the role of ASHAs, leading them to function predominantly as service providers rather than as more embedded health facilitators within their communities, as revealed by the recent study of Legare and colleagues [13]. Service delivery may have been prioritized because it relates to easily countable metrics: mothers either gave birth in a hospital or did not. Further, ASHAs and their clients are financially incentivized to achieve some of these outcomes, including institutionalized delivery.

The ASHA role involves service extension, linking hard-to-reach communities with the formal medical system. However, the intense focus on the uptake of specific services frequently overlooks the ASHA’s potential to leverage their intimate understanding of local customs and beliefs to bring about persuasive behavioral change and broader cultural change. The focus on the easily countable uptake of services narrows the frame of the program to those specific services. Thus, it follows to provide information about those services as the primary strategy for uptake. Sociocultural practices and processes have downstream consequences on service uptake, and changes in them should be revealed by service uptake metrics. However, you only look to measure the service uptake and not the sociocultural practices and processes. In that case, you miss the bigger picture and may limit the benefits of service uptake.

The official Indian government websites state that “capacity building of ASHA is being seen as a continuous process. ASHA will have to undergo a series of training episodes to acquire the necessary knowledge, skills, and confidence for performing her spelled-out roles.” (https://nhm.gov.in/index1.php?lang=1&level=3&sublinkid=184&lid=257) suggests that there is nothing precluding training that includes a focus on the social and cultural dynamics of decision-making. Ideally, experts in behavioral science methods and the local culture could collaborate to design this training [36]. It will be an essential step to realizing the envisioned roles of ASHAs as service providers, social mobilizers, and cultural facilitators. Of course, this research and translational work need to extend beyond Bihar to other regions of India.

There are multiple models of health behavior that could inform a more comprehensive training program for ASHAs. For example, the Health Belief Model (HBM) [38] represents a considerably more complex and nuanced framework for behavior change compared to the Deficit Model. HBM explicitly incorporates dimensions beyond the presence or absence of knowledge for explaining health decisions, such as perceived susceptibility, perceived severity, perceived benefits, perceived barriers, cues to action, and self-efficacy. Training ASHAs in this framework could facilitate ASHA interviews to identify each of these factors in their community and design new behavior change strategies tailored to this information. HBM is just one of several frameworks—such as the Cultural Ecology of Health—that advocate for a complex systems approach to understanding health in context [39].

## Conclusion

A lack of knowledge, health misconceptions, or health literacy is a big part of why someone does not take medical advice [40]. The Specific *Health* analyses show that ASHAs have more medical knowledge than their clients (and the full report offers further details). Transmitting medical and scientific knowledge will always be central to improving health decision-making, and research has demonstrated how best to explain medical science for motivating behavior [41,42]. However, even the ASHA’s health knowledge was incomplete; for example, it focused on the benefits of iron in the IFA tables (relating to blood health) but never mentioned the folic acid benefits related to congenital spinal disabilities. Calls to improve ASHA training should focus on medical knowledge in addition to social and cultural dynamics of decision-making. Even the most well-crafted explanation of some medical practice is transmitted in a social situation. Understanding how the social situation supports or hinders this transmission is critical in increasing the uptake of medical services. This understanding of the social setting needs to be one of the key focus areas for research and training for healthcare workers globally.

## Supporting information

S1 Table Complete list of vignette questions. (PDF)

S2 Table More example responses across response categories. (PDF)

S3 Table Proportion of responses for all eight vignettes, for Question 1 and 2, broek down by respondent and condition. (PDF)

S4 Table Supplementary GLMM information. (PDF)
