# Supplementary material for: Community health workers’ counseling is based on a deficit model of behavior change
Source: PLOS Glob Public Health. 2025 Jul 23;5(7):e0004167. doi: 10.1371/journal.pgph.0004167 (PMC12286350; doi:10.1371/journal.pgph.0004167)
Supplement: S1 Table — (PDF) [file pgph.0004167.s001.pdf]

S1 Table

Materials:

Complete list of vignette questions for Study 1.

Consistent Condition

1. Why did the mother choose to follow her ASHA’s recommendation?
2. Who helped the mother make the decision?
3. What do you think the ASHA said to persuade the mother to do the recommended behavior?
4. Was there any conflict between the mother and her family member?
5. How is that conflict resolved? Who initiates the resolution?
6. When should mothers be told about the recommendation on the perinatal journey?

Inconsistent Condition

1. Why did the mother not choose to follow her ASHA’s recommendation?
2. Who helped the mother make the decision?
3. What do you think the ASHA should have said to persuade the mother to do the recommended behavior?
4. Was there any conflict between the mother and her ASHA?
5. How is that conflict resolved? Who initiates the resolution?
6. When should mothers be told about the recommendation on the perinatal journey?
