# Supplementary material for: Community health workers’ counseling is based on a deficit model of behavior change
Source: PLOS Glob Public Health. 2025 Jul 23;5(7):e0004167. doi: 10.1371/journal.pgph.0004167 (PMC12286350; doi:10.1371/journal.pgph.0004167)
Supplement: S2 Table — (PDF) [file pgph.0004167.s002.pdf]

S2 Table

More Example Responses

Vignette	Question	Respondent	Condition	Code(s)	Response
Colostrum	1	ASHA	Consistent	Health/Bio (specific)	Anita fed colostrum to her child so that it does not have any diseases such as pneumonia, fever, turning body color blue.
Colostrum	3	ASHA	Inconsistent	Health/Bio	ASHA should have told Anita that colostrum is very beneficial for the child, it provides strength and helps in improving child's vision
IFA Tablet	1	ASHA	Consistent	Health/Bio (specific)<br>Knowledge/ Ignorance	The woman must have knowledge about it already that by having IFA tablets you have strength. There is no lack of blood. Anaemia is not there.
IFA Tablet	3	Mother	Consistent	Health/Bio	Blood loss can occur from having a baby in the stomach. Eating IFA creates blood. Rekha will get strength.
Exclusive Breastfeeding	1	Mother	Inconsistent	Social Dynamics	Meena gave her child water along with breastmilk because her family suggested to do so.
Exclusive Breastfeeding	3	Mother	Consistent	Health/Bio	Meena has agreed to only breastfeed the child because the mother should feed her milk only, this makes the child strong.
Hospital Birth	1	ASHA	Inconsistent	Health/Bio<br>Social Dynamics<br>Other Benefits & Costs<br>Knowledge/ Ignorance	Manju must have wondered what is the benefit of going to the hospital. She will not know that the child is born well in the hospital. ASHA would not have explained properly. Or it could also be that all the people in her house will have home delivery and would think that it costs more money in the hospital. There may also be family pressure, someone may have scared Manju that the nurse beats in the hospital. The child is not delivered fast. The male doctor also puts his hands on the patient and sees everything.

Hospital Birth	3	Mother	Consistent	Health/Bio<br>Other Benefits & Costs	Giving birth at hospital is good. Both mother and child would be good. Birth certificate is made. Child gets the B.C.G injection immediately. Gets money from Government also.
Family Planning 1	1	ASHA	Consistent	Health/Bio<br>Social Dynamics	Woman would have thought age is less now, it would be good to have child when grows a little. Age should be at least 21 to have children. Husband and wife would spend time nicely if child is not born now. Body will get weak if child is born in a young age
Family Planning 1	3	ASHA	Inconsistent	Health/Bio	If Ranjani would have got married in a young then ASHA should tell that she will get weak by having kids at a young age. Let yourself get a bit older then have children. This is how she should explain.
Family Planning 2	1	Mother	Inconsistent	Health/Bio<br>Other Benefits & Costs	Neetu must have thought that having more children will cause trouble to both mother and baby. If mother is not healthy then who will take care of the baby. There will be difficulty in catering to the child's care, studies, food and drink. The larger the family, the more difficulty will increase. A small family is a happy family. That's why Neetu decided to use birth control.
Family Planning 2	3	Mother	Inconsistent	Other Benefits & Costs	Neetu started using family planning measures because if there are less children at home, expenditure on education would be less. There would be no problems if we have less children.
Vaccination in pregnancy	1	ASHA	Consistent	Health/Bio (Specific)	Suman may have decided to take TT injection during pregnancy because the mother and child should be protected from tetanus disease. Child will not be handicapped.
Vaccination in pregnancy	3	Mother	Consistent	Health/Bio (Specific)	Suman thought good for her child. TT injection protects mother and child from

					tetanus. Both remains healthy. Mother do not suffer from any disease during pregnancy.
Vaccination Infancy	1	ASHA	Inconsistent	Health/Bio Knowledge/ Ignorance	Childs get fever by giving him vaccination.<br>What is the benefit of vaccination.. And also did not have knowledge.
Vaccination	3	Mother	Inconsistent	Health/Bio Social Dynamic	Soni's mother refuses to be vaccinated because the child will cry and become lame after being vaccinated. ASHA would tell that the child is not disabled by taking the vaccine, all the people in the village vaccinate their child. On saying this, Sony would have agreed to get her child vaccinated.
