# Supplementary material for: Community health workers’ counseling is based on a deficit model of behavior change
Source: PLOS Glob Public Health. 2025 Jul 23;5(7):e0004167. doi: 10.1371/journal.pgph.0004167 (PMC12286350; doi:10.1371/journal.pgph.0004167)
Supplement: S3 Table — (PDF) [file pgph.0004167.s003.pdf]

S3 Table

Question 1- Decision Reasons: Proportion of responses for all eight vignettes, broken down by respondent and condition.

Topic	Respondent	Condition	**Health - Biology**	**Social Dynamics**	**Other Benefits - Costs**	**Knowledge -Ignorance**
Colostrum	ASHA	Consistent	1.00	0.19	0.00	0.27
IFA	ASHA	Consistent	0.98	0.14	0.00	0.10
Exclusive Breastfeeding	ASHA	Consistent	0.86	0.19	0.00	0.02
Vaccines - Pregnancy	ASHA	Consistent	0.93	0.05	0.00	0.00
Vaccines – Infancy	ASHA	Consistent	1.00	0.00	0.00	0.00
Family Planning 1 - no children	ASHA	Consistent	0.77	0.27	0.18	0.00
Family Planning 2 - multiple children	ASHA	Consistent	0.23	0.15	0.92	0.00
Institutional Delivery	ASHA	Consistent	0.96	0.00	0.58	0.00
Colostrum	Mother	Consistent	0.92	0.14	0.00	0.01
IFA	Mother	Consistent	0.99	0.04	0.00	0.01
Exclusive Breastfeeding	Mother	Consistent	0.99	0.03	0.00	0.01
Vaccines - Pregnancy	Mother	Consistent	1.00	0.00	0.00	0.00
Vaccines – Infancy	Mother	Consistent	1.00	0.09	0.00	0.00
Family Planning 1 - no children	Mother	Consistent	0.85	0.11	0.11	0.00
Family Planning 2 - multiple children	Mother	Consistent	0.20	0.00	0.90	0.00
Institutional Delivery	Mother	Consistent	0.97	0.00	0.48	0.00
Colostrum	ASHA	Inconsistent	0.70	0.30	0.00	0.41
IFA	ASHA	Inconsistent	0.76	0.21	0.00	0.36
Exclusive Breastfeeding	ASHA	Inconsistent	0.80	0.15	0.00	0.34
Vaccines - Pregnancy	ASHA	Inconsistent	0.33	0.31	0.00	0.64
Vaccines – Infancy	ASHA	Inconsistent	0.85	0.15	0.04	0.31
Family Planning 1 - no children	ASHA	Inconsistent	0.52	0.64	0.00	0.23
Family Planning 2 - multiple children	ASHA	Inconsistent	0.25	0.71	0.68	0.21
Institutional Delivery	ASHA	Inconsistent	0.46	0.42	0.73	0.46

Colostrum	Mother	Inconsistent	0.81	0.13	0.00	0.20
IFA	Mother	Inconsistent	0.90	0.14	0.00	0.19
Exclusive Breastfeeding	Mother	Inconsistent	0.67	0.13	0.00	0.24
Vaccines - Pregnancy	Mother	Inconsistent	0.52	0.30	0.00	0.63
Vaccines – Infancy	Mother	Inconsistent	0.85	0.04	0.00	0.19
Family Planning 1 - no children	Mother	Inconsistent	0.41	0.63	0.44	0.11
Family Planning 2 - multiple children	Mother	Inconsistent	0.57	0.14	0.36	0.04
Institutional Delivery	Mother	Inconsistent	0.42	0.18	0.82	0.52

Question 2- Influence Methods: Proportion of responses for all eight vignettes, broken down by respondent and condition.

Topic	Respondent	Condition	Health - Biology	Social Dynamics	Other Benefits-Costs	Knowledge - Ignorance
Colostrum	ASHA	Consistent	1	0.58	0.15	0
IFA	ASHA	Consistent	1	0.05	0.05	0
Exclusive Breastfeeding	ASHA	Consistent	0.91	0.11	0.05	0
Vaccines - Pregnancy	ASHA	Consistent	0.98	0.1	0.07	0
Vaccines – Infancy	ASHA	Consistent	0.97	0.12	0	0
Family Planning 1 - no children	ASHA	Consistent	1	0.1	0.05	0.07
Family Planning 2 - multiple children	ASHA	Consistent	0.54	0.38	0.39	0
Institutional Delivery	ASHA	Consistent	1	0	0.62	0
Colostrum	Mother	Consistent	0.91	0.65	0	0
IFA	Mother	Consistent	0.97	0.1	0	0
Exclusive Breastfeeding	Mother	Consistent	0.89	0.04	0	0
Vaccines - Pregnancy	Mother	Consistent	1	0.01	0	0
Vaccines – Infancy	Mother	Consistent	0.99	0.04	0.07	0
Family Planning 1 - no children	Mother	Consistent	0.98	0.08	0.11	0
Family Planning 2 - multiple children	Mother	Consistent	0.5	0.06	0.91	0.03
Institutional Delivery	Mother	Consistent	0.96	0	0.58	0

Colostrum	ASHA	Inconsistent	0.96	0.08	0	0
IFA	ASHA	Inconsistent	0.98	0.1	0.07	0
Exclusive Breastfeeding	ASHA	Inconsistent	0.67	0.1	0.05	0
Vaccines - Pregnancy	ASHA	Inconsistent	0.98	0.12	0.02	0
Vaccines – Infancy	ASHA	Inconsistent	0.96	0.2	0	0
Family Planning 1 - no children	ASHA	Inconsistent	0.98	0	0.1	0
Family Planning 2 - multiple children	ASHA	Inconsistent	0.33	0.19	0.85	0.04
Institutional Delivery	ASHA	Inconsistent	0.88	0	0.85	0
Colostrum	Mother	Inconsistent	0.9	0.01	0	0
IFA	Mother	Inconsistent	0.94	0.03	0	0
Exclusive Breastfeeding	Mother	Inconsistent	0.93	0.01	0	0
Vaccines - Pregnancy	Mother	Inconsistent	0.9	0.05	0	0
Vaccines – Infancy	Mother	Inconsistent	1	0.03	0.02	0.03
Family Planning 1 - no children	Mother	Inconsistent	0.95	0.02	0.08	0
Family Planning 2 - multiple children	Mother	Inconsistent	0.32	0.24	0.89	0.05
Institutional Delivery	Mother	Inconsistent	0.91	0	0.66	0
