# Supplementary material for: Community health workers’ counseling is based on a deficit model of behavior change
Source: PLOS Glob Public Health. 2025 Jul 23;5(7):e0004167. doi: 10.1371/journal.pgph.0004167 (PMC12286350; doi:10.1371/journal.pgph.0004167)
Supplement: S4 Table — (PDF) [file pgph.0004167.s004.pdf]

S4 Table

Supplementary GLMM information
We note that for consistency across analyses and direct comparison across DV's, we report analyses with the same fixed effects for every DV. For some analyses, not every fixed factor improved model fit.

Question 1
Model: DV ~ 1 + Condition + Respondent + Condition:Respondent + (1 | ID) + (1 | Vignette)

DV: Health/Bio

Effect	Estimate	SE	exp(B)	z	p
(Intercept)	1.747	0.512	5.740	3.416	< .001
Consistent - Inconsistent	2.269	0.198	9.671	11.475	< .001
ASHA - Mother	-0.314	0.188	0.731	-1.668	0.095
Consistent - Inconsistent ✻ ASHA - Mother	0.106	0.337	1.112	0.315	0.753

DV: Specific Health/Bio

Effect	Estimate	SE	exp(B)	z	p
(Intercept)	-1.092	0.490	0.336	-2.23	0.026
Consistent - Inconsistent	0.792	0.134	2.207	5.93	< .001
ASHA - Mother	0.585	0.151	1.796	3.88	< .001
Consistent - Inconsistent ✻ ASHA - Mother	0.700	0.263	2.013	2.66	0.008

DV: Social Dynamics

Effect	Estimate	SE	exp(B)	z	p
(Intercept)	-2.152	0.189	0.116	-11.36	< .001
Consistent - Inconsistent	-1.163	0.172	0.312	-6.77	< .001
ASHA - Mother	0.808	0.189	2.244	4.28	< .001
Consistent - Inconsistent ✻ ASHA - Mother	0.686	0.336	1.986	2.04	0.041

DV: Other Benefits or Costs

Effect	Estimate	SE	exp(B)	z	p
(Intercept)	-3.583	1.345	0.0278	-2.66	0.008
Consistent - Inconsistent	-0.356	0.197	0.7005	-1.81	0.071
ASHA - Mother	0.209	0.206	1.2327	1.01	0.311
Consistent - Inconsistent ✻ ASHA - Mother	0.967	0.398	2.6291	2.43	0.015

DV: Knowledge or Ignorance

Effect	Estimate	SE	exp(B)	z	p
(Intercept)	-2.73	0.301	0.0653	-9.06	< .001
Consistent - Inconsistent	-3.45	0.338	0.0316	-10.22	< .001
ASHA - Mother	1.25	0.336	3.4737	3.71	< .001
Consistent - Inconsistent ✻ ASHA - Mother	1.90	0.663	6.7055	2.87	0.004

Question 2

Model: DV ~ 1 + Condition + Respondent + Condition:Respondent + (1 | ID) + (1 | Vignette)

DV: Health/Bio

Effect	Estimate	SE	exp(B)	z	p
(Intercept)	2.900	0.521	18.17	5.563	< .001
Consistent - Inconsistent	0.875	0.217	2.40	4.034	< .001
ASHA - Mother	0.193	0.223	1.21	0.866	0.387
Consistent - Inconsistent ✻ ASHA - Mother	0.473	0.427	1.61	1.108	0.268

DV: Social Dynamics

Effect	Estimate	SE	exp(B)	z	p
(Intercept)	-2.769	0.476	0.0627	-5.82	< .001
Consistent - Inconsistent	0.955	0.195	2.5980	4.90	< .001
ASHA - Mother	0.708	0.202	2.0296	3.51	< .001
Consistent - Inconsistent ✻ ASHA - Mother	-0.457	0.387	0.6329	-1.18	0.237

DV: Other Benefits or Costs

Effect	Estimate	SE	exp(B)	z	p
(Intercept)	-2.4270	0.829	0.0883	-2.928	0.003
Consistent - Inconsistent	-0.0446	0.208	0.9564	-0.215	0.830
ASHA - Mother	0.4502	0.220	1.5687	2.043	0.041
Consistent - Inconsistent ✻ ASHA - Mother	-0.2535	0.415	0.7760	-0.610	0.542

DV: Knowledge or Ignorance

Effect	Estimate	SE	exp(B)	z	p
(Intercept)	-6.3798	1.321	0.00170	-4.830	< .001

Effect	Estimate	SE	exp(B)	z	p
Consistent - Inconsistent	0.0762	0.720	1.07919	0.106	0.916
ASHA - Mother	0.1777	0.724	1.19442	0.246	0.806
Consistent - Inconsistent ✻ ASHA - Mother	2.1356	1.440	8.46181	1.484	0.138

Question 1 and 2

Model: DV ~ 1 + Condition + Respondent + Question + Condition:Question + Respondent:Question + (1 | ID) + (1 | Vignette)

DV: Health/Bio

Effect	Estimate	SE	exp(B)	z	p
(Intercept)	2.3099	0.495	10.073	4.664	< .001
Consistent - Inconsistent	1.5514	0.140	4.718	11.091	< .001
ASHA - Mother	-0.0541	0.155	0.947	-0.348	0.728
3 - 1	1.2163	0.141	3.375	8.637	< .001
Consistent - Inconsistent ✻ 3 - 1	-1.4182	0.258	0.242	-5.491	< .001
ASHA - Mother ✻ 3 - 1	0.5677	0.263	1.764	2.159	0.031

Simple effects of Question : Parameter estimates

Condition	contrast	Estimate	SE	exp(B)	z	p
Inconsistent	3 - 1	1.925	0.169	6.86	11.40	< .001
Consistent	3 - 1	0.507	0.211	1.66	2.40	0.016

Note. Simple effects are estimated keeping constant other independent variable(s) in the model

DV: Social Dynamic

Effect	Estimate	SE	exp(B)	z	p
(Intercept)	-2.315	0.218	0.0987	-10.62	< .001
Consistent - Inconsistent	-0.186	0.123	0.8299	-1.52	0.129
ASHA - Mother	0.625	0.138	1.8674	4.54	< .001
3 - 1	-0.383	0.122	0.6815	-3.14	0.002
Consistent - Inconsistent ✻ 3 - 1	2.071	0.242	7.9346	8.55	< .001
ASHA - Mother ✻ 3 - 1	-0.254	0.231	0.7757	-1.10	0.272

Simple effects of Question : Parameter estimates

Condition	contrast	Estimate	SE	exp(B)	z	p
Inconsistent	3 - 1	-1.419	0.175	0.242	-8.12	< .001
Consistent	3 - 1	0.652	0.169	1.920	3.86	< .001

Note. Simple effects are estimated keeping constant other independent variable(s) in the model

DV: Other Benefits or Costs

Effect	Estimate	SE	exp(B)	z	p
(Intercept)	-2.63506	0.917	0.0717	-2.8720	0.004
Consistent - Inconsistent	-0.25357	0.135	0.7760	-1.8814	0.060
ASHA - Mother	0.30505	0.159	1.3567	1.9196	0.055
3 - 1	-0.34550	0.141	0.7079	-2.4587	0.014
Consistent - Inconsistent ✻ 3 - 1	0.55151	0.265	1.7359	2.0837	0.037
ASHA - Mother ✻ 3 - 1	0.00722	0.279	1.0072	0.0258	0.979

Simple effects of Question : Parameter estimates

Condition	contrast	Estimate	SE	exp(B)	z	p
Inconsistent	3 - 1	-0.6213	0.194	0.537	-3.207	0.001
Consistent	3 - 1	-0.0698	0.192	0.933	-0.363	0.717

Note. Simple effects are estimated keeping constant other independent variable(s) in the model

Question 1 and 2 with Vignette Category

Model: DV ~ 1 + Question + Condition + Vignette Category + Question:Condition + Question:Vignette Category + Condition:Vignette Category + (1 | ID) + (1 | Vignette)

DV: Health/Bio

Effect	Estimate	SE	exp(B)	z	p
(Intercept)	2.0604	0.384	7.849	5.3635	< .001
3 - 1	1.0844	0.133	2.958	8.1499	< .001
Consistent - Inconsistent	1.5944	0.141	4.926	11.2850	< .001
Other Benefits - Health	-1.8555	0.759	0.156	-2.4438	0.015
3 - 1 ✻ Consistent - Inconsistent	-1.5664	0.263	0.209	-5.9618	< .001
3 - 1 ✻ Other Benefits - Health	0.8502	0.258	2.340	3.2999	< .001
Consistent - Inconsistent ✻ Other Benefits - Health	0.0202	0.278	1.020	0.0726	0.942

Simple effects of Question : Parameter estimates

Vignette Category	Condition	contrast	Estimate	SE	exp(B)	z	p
Health	Inconsistent	3 - 1	1.443	0.196	4.231	7.359	< .001
	Consistent	3 - 1	-0.124	0.259	0.883	-0.478	0.633
Other Benefits	Inconsistent	3 - 1	2.293	0.219	9.902	10.478	< .001
	Consistent	3 - 1	0.726	0.229	2.067	3.165	0.002

DV: Social Dynamics

Effect	Estimate	SE	exp(B)	z	p
(Intercept)	-2.47349	0.226	0.0843	-10.9241	< .001
3 - 1	-0.45888	0.126	0.6320	-3.6402	< .001
Consistent - Inconsistent	-0.35033	0.131	0.7045	-2.6752	0.007
Other Benefits - Health	0.00980	0.434	1.0099	0.0226	0.982
3 - 1 ✻ Consistent - Inconsistent	1.99225	0.244	7.3320	8.1703	< .001
3 - 1 ✻ Other Benefits - Health	-0.69011	0.254	0.5015	-2.7221	0.006
Consistent - Inconsistent ✻ Other Benefits - Health	-1.01852	0.259	0.3611	-3.9332	< .001

Simple effects of Question : Parameter estimates

Vignette Category	Condition	contrast	Estimate	SE	exp(B)	z	p
Health	Inconsistent	3 - 1	-1.110	0.201	0.330	-5.534	< .001
	Consistent	3 - 1	0.882	0.186	2.416	4.747	< .001
Other Benefits	Inconsistent	3 - 1	-1.800	0.231	0.165	-7.803	< .001
	Consistent	3 - 1	0.192	0.244	1.212	0.789	0.430

DV: Other Benefits or Costs

Effect	Estimate	SE	exp(B)	z	p
(Intercept)	-2.1504	0.353	0.116	-6.094	< .001
3 - 1	0.2097	0.207	1.233	1.016	0.310
Consistent - Inconsistent	0.0536	0.198	1.055	0.271	0.786
Other Benefits - Health	5.1176	0.705	166.939	7.260	< .001
3 - 1 ✻ Consistent - Inconsistent	0.5058	0.272	1.658	1.861	0.063
3 - 1 ✻ Other Benefits - Health	-1.5457	0.412	0.213	-3.748	< .001
Consistent - Inconsistent ✻ Other Benefits - Health	-0.9362	0.397	0.392	-2.359	0.018

Simple effects of Question : Parameter estimates

Vignette Category	Condition	contrast	Estimate	SE	exp(B)	z	p
Health	Inconsistent	3 - 1	0.730	0.419	2.074	1.74	0.082
	Consistent	3 - 1	1.236	0.399	3.440	3.09	0.002
Other Benefits	Inconsistent	3 - 1	-0.816	0.204	0.442	-4.01	< .001
	Consistent	3 - 1	-0.310	0.196	0.733	-1.58	0.113
